# High-Intensity Ultrasound Processing of Passion Fruit Pulp: Effects on Physicochemical Properties, Microbiological Quality, Bioactive Compound Retention, and Ascorbate Oxidase Activity

**DOI:** 10.3390/foods15071187

**Published:** 2026-04-01

**Authors:** Lorena Santos de Almeida, Fernanda Ribeiro Pitta Teixeira, Camila de Almeida Moreira, Joselene Conceição Nunes Nascimento, Luciano Almeida de Albuquerque, Mariana Nougalli Roselino, Jaciene Lopes de Jesus Assis, Ronielli Cardoso Reis, Onildo Nunes de Jesus, Fabio de Souza Dias, Alini Tinoco Fricks

**Affiliations:** 1 Programa de Pós-Graduação em Ciência de Alimentos, Universidade Federal da Bahia, Salvador 40170-115, BA, Brazil; almeidalorena@ufba.br (L.S.d.A.); joselene.conceicao@ufba.br (J.C.N.N.); bioalbuquerque@gmail.com (L.A.d.A.); 2 Departamento de Análises Bromatológicas, Faculdade de Farmácia, Universidade Federal da Bahia, Salvador 40170-115, BA, Brazil; fernandapitta@ufba.br (F.R.P.T.); moreiracamila010@gmail.com (C.d.A.M.); mariana.roselino@ufba.br (M.N.R.); 3 Embrapa Mandioca e Fruticultura, Cruz das Almas 44380-000, BA, Brazil; jaciene.jesus@embrapa.br (J.L.d.J.A.); ronielli.reis@embrapa.br (R.C.R.); onildo.nunes@embrapa.br (O.N.d.J.); 4 Programa de Pós-Graduação em Química, Universidade Federal da Bahia, Salvador 40170-115, BA, Brazil; fsdias@ufba.br

**Keywords:** *Passiflora edulis* Sims., yellow passion fruit, purple passion fruit, rheological parameters, phenolic compounds, ascorbic acid, microbiological quality

## Abstract

This study aimed to evaluate the effects of high-intensity ultrasound (40 W/5 min), applied with and without mild heating (59 °C and 23 °C), and of pasteurization (63 °C/30 min), on the physicochemical, rheological, and microbiological parameters, as well as on ascorbate oxidase activity, total carotenoid content, phenolic compound profile, and antioxidant capacity of passion fruit (*Passiflora edulis* Sims.) pulps. Ultrasound processing induced changes in color (L*, a*, and b*), resulting in high ∆E values. Following ultrasound treatment, an increase in apparent viscosity at 100 s^−1^ was observed. Ultrasound also promoted partial inactivation of ascorbate oxidase and a significant reduction in mold and yeast counts. Moreover, the application of ultrasound without heating (US-20) promoted the retention of 55% of ascorbic acid after 63 days of storage. The condition with heating (US-60) led to an increase in catechin content in both bright red passion fruit pulp (173.96%) and yellow passion fruit pulp (5.89%), demonstrating a balance between the retention of bioactive compounds, microbial inactivation, and reduction in ascorbate oxidase activity. Therefore, these results highlight ultrasound as a non-thermal and sustainable technology capable of extending shelf life, maximizing the preservation of bioactive compounds, and enhancing the functional properties of fruit pulps.

## 1. Introduction

Passion fruit (*Passiflora edulis* Sims.), also known as “sour passion fruit,” is a fruit native to Brazil, traditionally recognized for its therapeutic and functional properties [1]. This species accounts for approximately 90% of global passion fruit production, establishing itself as the main economically relevant representative within the *Passiflora* genus [2]. In 2024, Brazil produced approximately 736.5 thousand tons of passion fruit across 47 thousand hectares, generating US$ 416 million in revenue for farmers [3]. Its fruits possess an aromatic pulp with a gelatinous texture and an intense yellow color, while the peel can display variations ranging from yellow to purple or deep violet hues [4,5].

Although yellow passion fruit (*Passiflora edulis* Sims.) is the most widely cultivated commercially, studies indicate that purple passion fruit (*Passiflora edulis* Sims.) may also represent an attractive alternative for the food market [6]. Despite belonging to the same species, purple-skinned fruits exhibit distinct characteristics compared with yellow-skinned ones, such as a sweeter pulp, smaller size, thinner peel, and purplish coloration, the latter attributed to the presence of anthocyanins [5,7].

Fruits of *Passiflora edulis* Sims. are widely known for their nutritional quality and the high consumer acceptance of their sensory profile. Furthermore, the pulp of this species constitutes a relevant source of compounds with high antioxidant capacity, such as vitamin C (ascorbic acid), carotenoids, and phenolic compounds [8,9,10]. These compounds play an essential role in neutralizing or eliminating free radicals, thereby preventing cellular oxidative damage [11]. A recent study reported that the bioactive compounds present in *Passiflora edulis* Sims. pulp extract may exhibit biological effects, including in vitro inhibition of enzymes involved in lipid metabolism modulation, as well as ex vivo promotion of vasodilation in rat aortic rings [12].

Passion fruit pulp is commonly marketed in its natural form; however, it also exhibits broad technological versatility, being used in the formulation of various food products, such as jams, juices, cookies, ice creams, and others [13]. One of the main challenges in the food industry is to develop techniques capable of reducing microbial activity and the action of enzymes responsible for food spoilage, without compromising the preservation of bioactive compounds [14,15]. Currently, pasteurization is the conventional method adopted by the food industry for processing fruit pulp [16]. In particular, Low Temperature Long Time (LTLT) pasteurization is frequently studied due to its operational simplicity, low cost, and effectiveness in eliminating pathogens [17]. However, its major limitation lies in the use of heat, which promotes the oxidation of thermosensitive molecules and leads to losses in the nutritional and sensory quality of juices and fruit pulps [15,18].

As an alternative, high-intensity ultrasound (HIUS) has emerged as an effective technique for processing and maintaining the quality of fruit juices and pulps [16,19,20]. It is a non-thermal, environmentally friendly method characterized by shorter processing times and energy savings compared with traditional techniques. HIUS is generated by a transducer that converts electrical energy into sound energy. Microbial and enzymatic inactivation through sonication is associated with two mechanisms: (i) physical, including cavitation and mechanical effects, and (ii) chemical, involving the formation of free radicals [21]. These mechanisms can cause destabilization of microbial cell membranes and structural alterations in enzymes, favoring their inactivation [15]. Additionally, HIUS promotes pore formation in plant cell membranes, allowing mass transfer to the extracellular medium [21,22].

Several studies have reported positive outcomes of thermosonication in the processing of fruit juices and pulps, particularly in the inactivation of pathogens and spoilage enzymes, while also contributing to the preservation of bioactive compounds and inducing changes in physical properties [18,23,24,25]. This technique combines the mechanical action of ultrasonic waves with the mild heating generated by the dissipation of acoustic energy during HIUS, resulting in a synergistic effect that enhances its technological efficiency [21].

The effect of HIUS processing on gulupa passion fruit pulp (*Passiflora edulis* f. *edulis*) was reported by Calderón-Martínez et al. [26]. The authors applied frequencies of 30 and 40 kHz for 10, 20, and 30 min and observed that the treatments increased Trolox Equivalent Antioxidant Capacity (TEAC) values and ascorbic acid content, while significantly reducing yeast counts during the storage period of gulupa pulp. Araújo et al. [15], when evaluating araçá-boi pulp (*Eugenia stipitata*), demonstrated that HIUS processing was effective in inactivating polyphenol oxidase (PPO), showing superior performance compared with LTLT pasteurization (5 min/70 °C).

However, studies that comparatively evaluate the efficiency of HIUS, with and without mild heating, on the physicochemical, microbiological, enzymatic, and bioactive compound properties of such matrices remain limited.

Therefore, the objective of this study was to evaluate the effects of HIUS, applied with and without mild heating, and of pasteurization on the physical and chemical characteristics, color attributes, bioactive compound concentration, antioxidant properties, ascorbate oxidase (ASO) activity, and microbiological quality of passion fruit (*Passiflora edulis* Sims.) pulps.

## 2. Materials and Methods

### 2.1. Plant Material and Pulp Preparation

Yellow passion fruits (*Passiflora edulis* Sims.) were purchased from local markets in the city of Simões Filho, Bahia, Brazil. Additionally, fruits from two genotypes selected by Embrapa Mandioca e Fruticultura were collected in the city of Cruz das Almas, Bahia, Brazil. These genotypes originated from intraspecific crosses (*P. edulis* × *P. edulis*), selected based on pulp yield, lower acidity, and higher soluble solids content. The selected hybrids, named “Bright Red Passion Fruit,” have a reddish-purple peel coloration associated with the presence of anthocyanins [5,7].

Approximately 15 kg of fruit from each variety were selected at the ripe stage of maturity and were free from skin cuts, bruised areas, or any visible deformities. After selection, the fruits were sanitized in a 200 mg/L sodium hypochlorite solution for 15 min and pulped using a stainless-steel sieve.

### 2.2. High-Intensity Ultrasound Processing and Pasteurization Parameters

Ultrasound processing was carried out using a 20 kHz ultrasonic processor equipped with a 13 mm probe (model Vibra-Cell VCX-500, Sonics & Materials, Newtown, CT, USA). Aliquots of 50 mL of pulp were placed in glass beakers and treated with HIUS for 5 min in pulsed mode (30 s on/30 s off) at 50% amplitude, reaching temperatures of 20 ± 2 °C (US-20) and 60 ± 3 °C (US-60). Due to the heat generated during sonication, an ice bath was used to prevent temperature rise in the US-20 samples. The probe was immersed approximately 25 mm deep in the sample. The ultrasonic intensity and energy density parameters used in the treatments are presented in Table 1.

To obtain pasteurized passion fruit pulp (50 mL), the Low Temperature Long Time (LTLT) thermal treatment method was employed using a water bath. The samples were maintained at 63 °C for 30 min (PS-63). Subsequently, the samples were immediately cooled in an ice bath until they reached a temperature of 7 °C, following the procedure described by Sanchez et al. [17]. Temperature monitoring during processing was performed using a thermometer.

Untreated samples were used as experimental controls. After processing, all pulps were stored in amber polypropylene bottles and kept in a freezer at −18 ± 1 °C for further analyses. All analyses were performed in biological triplicate.

### 2.3. pH, Soluble Solids, and Total Titratable Acidity

The pH of the passion fruit pulp was measured using a digital pH meter (model PHS3BW, Bel Engineering, Monza, Italy). After calibration, the electrode was immersed directly in the pulp at room temperature. Soluble solids were determined using a refractometer (model HI96800, Hanna Instruments, Woonsocket, RI, USA) at 25 °C, and the results were expressed in °Brix. For total titratable acidity, 5 g of passion fruit pulp were diluted in 40 mL of distilled water, and the samples were titrated with 0.1 M NaOH using 1% phenolphthalein as an indicator. The results were expressed as a percentage of citric acid.

### 2.4. Color Parameters

The color of the passion fruit pulp was determined using a colorimeter (model CR-410, Konica Minolta, Tokyo, Japan). The illuminant type was D65, and the degree of the observer was 2°. To measure color changes, the following parameters were determined: L* (lightness), a* (red/green coordinate), b* (yellow/blue coordinate), hue angle (h*) and chroma (C*), according to the Commission Internationale de l’Éclairage (CIE). Based on the L*, a*, and b* coordinate values, the parameters related to color difference (ΔE) and browning index (BI) were calculated according to Equations (1) and (2) [17,27].

(1) $$∆E=\sqrt{(L^{*}-L_{0}^{*}{)}^{2}+(a^{*}-a_{0}^{*}{)}^{2}+(b^{*}-b_{0}^{*}{)}^{2}}$$

where $L_{0}^{*}$, $a_{0}^{*}$ and $b_{0}^{*}$ correspond to the color parameters of the control sample.

(2) $$BI=\frac{\left[100(X-0.31)\right]}{0.172}$$

where

$$X=\frac{a^{*}+0.75\times L^{*}}{5.645\times L^{*}+a^{*}-3.012\times b^{*}}$$

### 2.5. Cloud Stability and Centrifugal Sedimentation

Cloud stability and sedimentation were determined according to the methodology described by Shen et al. [28], with modifications. Aliquots of 10 g of passion fruit pulp were centrifuged (model CR22N, Himac, Kanagawa, Japan) at 4200× *g* for 15 min at room temperature. The absorbance of the supernatant was then measured using a spectrophotometer (model UV-1800, Shimadzu, Kyoto, Japan) at 625 nm. Cloud stability (*CS*) was calculated according to Equation (3):

(3) CS(%)=(CA/C0)×100

where C0 is the absorbance before centrifugation and CA is the absorbance after centrifugation.

For the determination of centrifugal sedimentation, 10 g samples of passion fruit pulp were centrifuged (model CR22N, Himac, Kanagawa, Japan) at 3500× *g* for 15 min at room temperature. The supernatant was discarded, and the sediment obtained was weighed. The centrifugal sedimentation (*SR*) rate was calculated using Equation (4):

(4) SR(%)=(m2/m1)×100

where m1 is the mass of the pulp before centrifugation and m2 is the mass of the precipitate after centrifugation.

### 2.6. Rheological Characteristics

The rheological characteristics of the pulp were determined using a rotational rheometer with concentric cylinders (model Rheotest 2.1, Medingen, Haake, Nordrhein-Westfalen, Germany) at 25 °C. The flow curves of the samples were obtained at shear rates ranging from 0.56 to 243 rpm. Data were fitted using the Ostwald–de Waele (Power Law) model, which defines the relationship between shear stress (*σ*) and shear rate (*γ*) according to Equation (5):

(5) $$\sigma =K\gamma^{\left(n\right)}$$

where σ is the shear stress (Pa), *K* is the consistency index (Pa.s ^n^), *γ* is the shear rate (s^−1^) and n is the flow behavior index (dimensionless). This model was used to evaluate the pseudoplastic behavior of each passion fruit pulp sample.

Rheological data were fitted to the Ostwald–de Waele model to obtain apparent viscosity parameters based on ascending shear rate curves within the range of 25 to 1000 s^−1^. Apparent viscosity was calculated using Equation (6):

(6) $$\mu =K\gamma^{\left(n-1\right)}$$

where *μ* is the apparent viscosity, *K* is the consistency index, *γ* is the shear rate and n is the flow behavior index. The results were expressed in mPa·s.

### 2.7. Determination of Ascorbic Acid

Ascorbic acid content was determined according to the method described by Oliveira [29]. Initially, 5 g of passion fruit pulp were homogenized with a 0.4% oxalic acid solution, and the volume was brought to 25 mL in a volumetric flask to stabilize the ascorbic acid. The mixture was then filtered through filter paper. Subsequently, a 1 mL aliquot of the filtrate was transferred to a test tube containing 9 mL of a 3% DCFI solution (2,6-dichlorophenolindophenol). The absorbance of the solution was determined at 520 nm using a spectrophotometer (UV-1800, Shimadzu, Kyoto, Japan). After the spectrophotometric reading, a crystal of ascorbic acid was added to the mixture, promoting the reduction of DCFI and the consequent discoloration of the dye, a procedure used to confirm the reactivity of the reagent and the redox nature of the reaction. The absorbance of the solution was then measured again at 520 nm under the same analytical conditions. The quantification of ascorbic acid was performed using an analytical calibration curve, and the results were expressed as mg 100 g^−1^. The ascorbic acid content of the pulp stored at −18 ± 0.1 °C was monitored every 7 days over a period of 63 days. The residual mean of ascorbic acid throughout storage was calculated according to Equation (7):

(7) $$Residual\;mean\left(\%\right)=100\times \frac{M_{t}}{M_{0}}$$

where $M_{t}$ is the ascorbic acid content (mg 100 g^−1^) of the treated sample and $M_{0}$ is the ascorbic acid content (mg 100 g^−1^) of the control sample.

### 2.8. Ascorbate Oxidase Activity

Ascorbate oxidase (ASO) activity was determined following the procedure described by Cardello and Cardello [30], with modifications. Samples of 2 g of passion fruit pulp were diluted in 20 mL of 0.1 M sodium phosphate buffer (pH 5.5). The extract was homogenized and centrifuged (model CR22N, Himac, Kanagawa, Japan) at 15,000× *g* for 30 min at 4 °C, and the supernatant was collected. For the assay, 160 µL of extract was incubated with 400 µL of 0.1 M sodium phosphate buffer (pH 5.5) and 400 µL of 1.0 mM ascorbic acid solution at room temperature. The reaction was stopped by adding 2.4 mL of 0.2 N hydrochloric acid every 30 s for 3 min, totaling seven readings. Absorbance was measured at 244 nm using a spectrophotometer (model UV-1800, Shimadzu, Kyoto, Japan). The residual enzyme activity of the stored pulp (−18 ± 0.1 °C) was monitored over a 63-day period. The residual activity was calculated according to Equation (8):

(8) $$Residual\;Activity\left(\%\right)=100\times \frac{A_{t}}{A_{0}}$$

where $A_{t}$ is the ASO activity (U/g) of the treated sample and $A_{0}$ is the ASO activity (U/g) of the control sample.

### 2.9. Determination of Total Carotenoids

The determination of total carotenoids (TC) was performed according to the methodology described by Rodriguez-Amaya and Kimura [31]. Approximately 5 g of passion fruit pulp were weighed and homogenized using a porcelain pestle with 5 g of Celite 545 and about 100 mL of acetone for the extraction step. The acetone extract was then filtered under vacuum and transferred to a 500 mL separatory funnel containing 30–40 mL of hexane and 15 mL of distilled water. Subsequently, 300 mL of a 0.41% saline solution were added to remove residual acetone, and the mixture was allowed to stand for 15 min. This washing step was repeated three times. The filtrate was passed through cotton covered with anhydrous sodium sulfate, and the extract was collected in an amber volumetric flask (25 or 50 mL), with the volume completed using hexane. Absorbance was measured in a spectrophotometer (UV-1800, Shimadzu, Kyoto, Japan) at 450 nm, and the results were expressed as µg/g of β-carotene (fresh weight basis) and at 470 nm for results expressed as µg g^−1^ of lycopene (fresh weight basis). The TC content was calculated according to Equation (9):

(9) $$TC(µg/g)=\frac{\left(A\times V\times 10^{4}\right)}{A_{1\mathrm{cm}}\times W}$$

where *A* is the measured absorbance, *V* is the final extract volume (25 or 50 mL), *A*_1__cm_ is the absorption coefficient for β-carotene (2592) and lycopene (3450), and W is the sample weight (g).

### 2.10. Total Phenolic Content and Antioxidant Capacity

#### 2.10.1. Extraction Procedure

The extract was obtained according to the methodology described by Larrauri et al. [32], with modifications. Five grams of sample were diluted in 15 mL of a methanol–distilled water solution (50%). The mixture was homogenized and subjected to an ultrasonic bath at 25 kHz (model USC-1800, Unique, São Paulo, Brazil) for 20 min at room temperature. After extraction, the mixture was centrifuged (model CR22N, Himac, Kanagawa, Japan) at 15,000 rpm for 15 min at room temperature, and the first supernatant was collected. The residue was then resuspended in 15 mL of 70% acetone solution, re-extracted, and centrifuged again, after which the second supernatant was collected. Both supernatants were transferred to an amber volumetric flask (50 mL), the volume was adjusted with distilled water, and the extract was stored at −18 °C. The obtained extract was used to determine the total extractable polyphenol content and antioxidant capacity.

#### 2.10.2. Determination of Total Phenolic Content

The determination of total phenolic content (TPC) was performed according to the method described by Obanda et al. [33], with adaptations. For the assay, 1 mL of the hydro-methanolic extract was mixed with 1 mL of 25% Folin–Ciocalteu reagent, 2 mL of 20% sodium carbonate, and 2 mL of distilled water, followed by incubation for 30 min in the dark at room temperature. After the reaction period, absorbance was measured at 700 nm using a UV–Vis spectrophotometer (UV-1800, Shimadzu, Kyoto, Japan). The results were expressed as mg gallic acid equivalents (GAE) per gram of sample (mg GAE g^−1^).

#### 2.10.3. DPPH Radical Scavenging Assay

The DPPH radical scavenging assay was conducted following the method described by Rufino et al. [34], with modifications. For the assay, 100 μL of extract was added to 3.9 mL of 0.06 mM DPPH solution, adjusted to an initial absorbance below 0.70. The mixture was incubated in the dark for 1 h at room temperature. After incubation, absorbance was recorded at 517 nm using a spectrophotometer (UV-1800, Shimadzu, Kyoto, Japan). Results were expressed as µM Trolox equivalents (TE) per gram of sample (µM TE g^−1^).

#### 2.10.4. ABTS^+^ Radical Cation Scavenging Assay

The ABTS^+^ [2,2′-azino-bis(3-ethylbenzothiazoline-6-sulfonic acid)] radical cation scavenging assay was carried out according to the method described by Rufino et al. [34] with modifications. The ABTS^+^ radical cation was generated by reacting 5 mL of 7.7 mM ABTS solution with 88 μL of 140 mM potassium persulfate, followed by incubation for 16 h in the dark at room temperature. After the reaction, the radical solution was diluted with distilled water to obtain an absorbance of 0.70 ± 0.02 at 734 nm, as measured in a spectrophotometer (UV-1800, Shimadzu, Kyoto, Japan). For the assay, 30 μL of extract was added to 3 mL of the diluted ABTS^+^ solution and incubated for 6 min at room temperature. After the reaction, absorbance was measured, and the results were expressed as µM TE per gram of sample (µM TE g^−1^).

### 2.11. Quantification of Phenolic Content

The hydroethanolic extract was prepared according to the method described by De Carvalho et al. [35], with adaptations. Briefly, 2 g of freeze-dried passion fruit pulp were added to 10 mL of 70% ethanol extraction solution. The mixture was homogenized and subjected to an ultrasonic bath at 25 kHz (model USC-1800, Unique, São Paulo, Brazil) for 20 min at room temperature. After extraction, the mixture was centrifuged (model CR22N, Himac, Kanagawa, Japan) at 4000 rpm for 15 min at 4 °C. The supernatant was collected and brought to a final volume of 10 mL in a volumetric flask. The extract was stored at −80 °C until chromatographic analysis. On the day of analysis, an aliquot (500 μL) of the extract was filtered through a 0.45 μm HV Millex filter unit and diluted with 500 μL of 70% ethanol. Phenolic compounds were quantified according to the method described by de Albuquerque et al. [36]. Analyses were performed using a high-performance liquid chromatography (HPLC) system (model 1290, Agilent Technologies, Santa Clara, CA, USA) equipped with a quaternary pump, autosampler (model ASI-100, Supelco, Bellefonte, PA, USA), and a UV–visible diode array detector (DAD). Sample separation was achieved using a reverse-phase C18 HPLC column (ZORBAX ODS, Agilent Technologies, Santa Clara, CA, USA), with dimensions of 25 cm × 4.6 mm × 5 μm. The mobile phase consisted of solvent A (water/methanol/acetic acid, 93:5:2) and solvent B (methanol/water/acetic acid, 88:10:2), both pre-filtered under vacuum using quantitative filter paper (7.0 cm diameter, 80 g/m^2^) and sonicated for 20 min in an ultrasonic bath to remove dissolved gases. Chromatographic separation was carried out under gradient conditions using 70% solvent A and 30% solvent B, with a flow rate of 1.0 mL/min for 70 min. The injection volume was 20 μL. Chromatograms were recorded at wavelengths of 260, 272, 280, 310, and 330 nm. Standard calibration curves were prepared using concentrations of 0.2, 0.6, 1.0, 1.4, 1.8, 2.2, 2.6, 3.0, 3.4, 3.8, 4.2, 4.6, and 5.0 mg/mL with standard solutions of catechin (R^2^ 0.998), vanillic acid (R^2^ 0.992), gallic acid (R^2^ 0.999), syringic acid (R^2^ 0.999), caffeic acid (R^2^ 0.996), chlorogenic acid (R^2^ 0.998), ferulic acid (R^2^ 0.994), and synaptic acid (R^2^ 0.995). Identification of phenolic compounds was based on retention times and UV–Vis spectra compared to those of authentic standards, using EZChrom software. Results were expressed as µg per 100 g dry weight (DW) of pulp (µg 100^−1^ DW).

### 2.12. Microbiological Analysis

Pulp samples were analyzed for counts of mesophilic aerobic bacteria, molds and yeasts, and for the determination of the Most Probable Number (MPN) of total and thermotolerant coliforms, *Escherichia coli*, and the presence of *Salmonella* spp., according to analytical methods established by the American Public Health Association (APHA) [37]. Aliquots of 1 mL of pulp were serially diluted (10^0^, 10^−1^, and 10^−2^) in 0.1% peptone water and homogenized using a vortex mixer (Phoenix, São Paulo, Brazil) for two minutes at normal speed. For the enumeration of mesophilic aerobic bacteria, 1 mL of each dilution was pour-plated in Plate Count Agar (PCA) (Difco™, Franklin Lakes, NJ, USA) and incubated in an inverted position in a bacteriological incubator at 35 °C for 48 h. Mold and yeast counts were determined by surface plating: 0.1 mL of each dilution was spread on Dichloran Rose Bengal Chloramphenicol Agar (DRBC) (KASVI, Paraná, Brazil) using a Drigalski spatula. Plates were incubated without inversion in a B.O.D. chamber at 25 °C for 5 days. Colony counts were performed using a colony counter (model CP 600 Plus, Phoenix^®^, São Paulo, Brazil), and results were expressed as log CFU/mL. Determination of total and thermotolerant coliforms was carried out using the multiple-tube MPN technique. For total coliform testing, 1 mL of each dilution was inoculated into tubes containing 10 mL of Lauryl Sulfate Tryptose Broth (LST) (KASVI, Paraná, Brazil) and incubated at 35 °C for 48 h. Tubes showing gas production in Durham tubes were considered presumptively positive and subjected to confirmatory testing for total and thermotolerant coliforms. A loopful from positive tubes was inoculated into Brilliant Green Bile Broth (KASVI, Paraná, Brazil) and incubated at 35 °C for 48 h for total coliform confirmation, and into EC Broth (KASVI, Paraná, Brazil) and incubated at 44.5 °C for 48 h in a water bath for thermotolerant coliforms. Gas production in Durham tubes confirmed the presence of total coliforms, and results were expressed as MPN/mL. Positive tubes for thermotolerant coliforms were streaked on Eosin Methylene Blue Agar (EMB) using the depletion streaking technique. However, no colonies exhibited the typical metallic green sheen characteristic of *Escherichia coli*, eliminating the need for biochemical confirmation. Results were also expressed as MPN/mL. Detection of *Salmonella* spp. was performed using pre-enrichment in Simple Lactose Broth (SLB) (Merck KGaA, Hessen, Germany), where 10 mL of sample were inoculated into 90 mL of SLB and incubated at 36 °C for 24 h. Selective enrichment was then performed in Rappaport–Vassiliadis (RV) and Tetrathionate (TT) broths (Merck KGaA, Hessen, Germany), followed by selective differential plating. A loopful from the enriched culture was streaked on Hektoen Enteric Agar (HE), Salmonella–Shigella Agar (SS), and Xylose Lysine Deoxycholate Agar (XLD) (Difco™, NJ, USA). Plates were incubated at 36 °C for 24 h, and results were expressed as absence in 25 mL.

### 2.13. Statistical Analysis

Mean values of all variables were subjected to Principal Component Analysis (PCA). The variables included pH, soluble solids (SS), total acidity, color coordinates L*, a*, and b*, sedimentation ratio (SR), cloud stability (CS), rheological parameters K, n, and apparent viscosity at 100 s^−1^, ascorbic acid (AA) content at 0, 35, and 63 days, ascorbate oxidase (ASO) activity at 0, 35, and 63 days, total carotenoids (TC) expressed as β-carotene and lycopene, total phenolic compounds (TPC), DPPH, ABTS, catechin, vanillic acid, gallic acid, syringic acid, caffeic acid, chlorogenic acid, ferulic acid, synaptic acid, mesophilic aerobic bacteria, and molds and yeasts. Triplicate analyses were maintained, and the dataset was preprocessed using autoscaling. PCA was conducted in R version 4.5.1 (https://www.r-project.org/) using the FactoMineR and Factoextra packages (accessed on 2 November 2025).

All analyses were performed in triplicate, and the results were expressed as mean ± standard deviation. Data were subjected to analysis of variance (ANOVA), and mean comparisons were carried out using Tukey’s test at a 5% significance level (*p* ≤ 0.05), with Statistica software version 12.0.37 [38].

## 3. Results and Discussion

### 3.1. pH, Soluble Solids, and Total Acidity

Organic acids and sugars are basic constituents of passion fruit pulp and together determine not only its organoleptic characteristics and flavor profile, but are also key indicators for assessing the ripening stage of the fruit [39]. Table 2 presents the pH, soluble solids, and total acidity values of the bright red passion fruit and yellow passion fruit pulps (*Passiflora edulis* Sims.).

As observed, the *Passiflora edulis* Sims. varieties exhibited a significant increase in pH after the US-60, US-20, and PS-63 treatments (*p* < 0.05). In the bright red passion fruit variety, the pH increased slightly from 2.66 to 2.71–2.73, while in the yellow passion fruit variety, the pH increased from 2.44 to 2.75–2.79. Post-processing changes were also observed in the soluble solids content of passion fruit pulps treated by HIUS with mild heating (US-60) (*p* < 0.05). In the bright red passion fruit pulp, HIUS treatment (5 min/60 °C) resulted in a 5.5% increase in soluble solids relative to the control sample. In the yellow passion fruit pulp, the increase was 4.67% compared with the control sample.

Pratap-Singh and Mandal [40] reported that the pH of watermelon juice treated in a continuous-flow pulsed UV light reactor varied only slightly, ranging from 5.04 to 5.16. The authors suggested that the processing intensity applied was not sufficient to significantly alter the ionic equilibrium of the watermelon juice. This scenario may explain the slight increase in the pH value observed in the evaluated passion fruit pulp.

The increase in soluble solids content in the processed *Passiflora edulis* Sims. pulp can be explained by the hydrolysis of polysaccharides and the significant evaporation of water, processes that may occur during HIUS processing through acoustic cavitation, as well as during intense thermal processing (pasteurization) [16,41,42]. Changes in soluble solids have also been reported in recent studies by other authors. Araújo et al. [15] evaluated the effect of HIUS at 19 kHz with an energy density of 7000 J g^−1^ on araçá-boi pulp and observed a 1.56% reduction in pH and a 16% increase in soluble solids compared with the untreated sample. Similar results were reported by Xing et al. [25], who observed a decrease in pH and an increase in soluble solids in strawberry pulp treated with pulsed HIUS (5 s on, 5 s off).

The processing conditions applied did not produce a significant effect on the total acidity of the passion fruit pulps evaluated (*p* > 0.05), with mean values of 4.01% and 4.09% for the bright red and yellow passion fruit pulps, respectively. Sweetness is a highly valued attribute that plays a key role in fruit consumption. The combination of a high soluble solids content and low acidity imparts a perception of a sweeter flavor to the fruit [5].

In this study, it was possible to observe that the bright red passion fruit pulp presents attributes that suggest greater sweetness. Changes related to the total acidity of the pulp occur due to a succession of biochemical processes such as the hydrolysis and oxidation of compounds. However, given the non-destructive nature of HIUS, it is evident that no alterations occurred in covalent bonds, thus preserving the initial acidity of the passion fruit pulp [43].

### 3.2. Color Parameters

Color is a direct indicator of fruit freshness and quality, a characteristic that strongly influences consumer satisfaction. Changes in coloration serve as an ideal marker of the biosynthesis and/or degradation of phytochemicals responsible for fruit pigmentation [44,45,46]. Table 2 presents the influence of HIUS and thermal processing on the color parameters of passion fruit pulp (*Passiflora edulis* Sims.).

As observed, the bright red passion fruit (*Passiflora edulis* Sims.) pulp exhibited an increase in L* (lightness) values after the treatments (*p* < 0.05), particularly in the sample processed with mild heating (US-60), which showed the highest brightness (48.33 ± 0.57). The chroma (C*) and hue angle (h*) parameters showed no change after HIUS processing with mild heating (US-60) (*p* > 0.05). An increase in yellow color intensity was observed in the treated pulps, as indicated by the b* coordinate (blueness/yellowness). This characteristic may be associated with the release of intracellular compounds, such as carotenoids, which impart the yellow coloration to *Passiflora edulis* Sims. pulp [47].

The increase in L* and a* parameters in the US-60 sample of bright red passion fruit pulp aligns with the findings of Shen et al. [28] in typical apple juice treated with HIUS at 60 °C for 12 min, where increases in L* and a* were primarily attributed to temperature elevation, resulting in improved color characteristics of the juice.

Similarly, temperature elevation also resulted in an increase in the L* parameter of the yellow passion fruit (*Passiflora edulis* Sims.) pulp in the PS-63 sample (51.69 ± 0.49) (*p* < 0.05). However, the chroma (C*) and hue angle (h*) parameters were not statistically influenced by the HIUS treatment with mild heating (US-60) (*p* > 0.05). The a* and b* coordinates decreased substantially in the PS-63 (12.57 ± 0.24 and 18.06 ± 0.18, respectively). This result indicates that the PS-63 yellow passion fruit pulp became lighter but less yellow and relatively greener.

**Figure 1:**
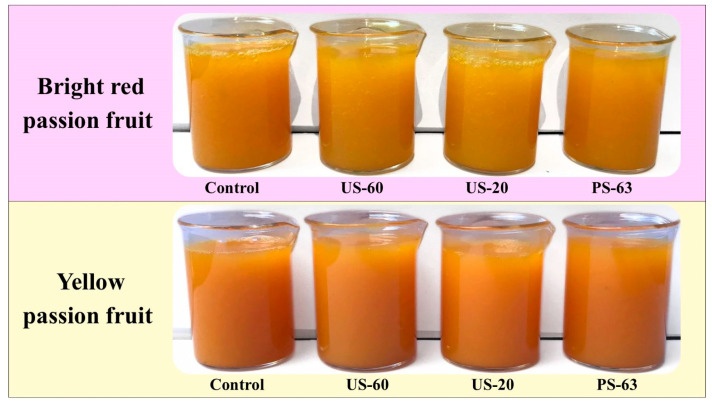
Effect of HIUS processing and thermal treatment on the visual appearance of bright red and yellow passion fruit pulps.

Total color difference (∆E) is a widely used parameter to quantify global variations in the coloration of fruits and their derivatives. A visually perceptible change is considered to occur when ∆E is equal to or greater than 1.5 [39,48]. As presented in Table 2, the treatments resulted in different intensities of color variation (∆E), using the control sample as a reference. Under white lighting and frontal view, the visual appearance of the bright red and yellow passion fruit pulps can be observed in Figure 1.

Color changes, indicated by ∆E values, were reported in all treatments, and it was possible to visually distinguish the treated samples, except for the US-20 treatment applied to the yellow passion fruit pulp, in which the visual difference was barely noticeable. Preservation of coloration may be mainly attributed to the maintenance of the initial temperature during HIUS processing, a condition that likely preserved thermolabile pigments and minimized non-enzymatic browning reactions such as the Maillard reaction [18]. In response to prolonged thermal treatment, the PS-63 yellow passion fruit pulp showed the highest ∆E value (10.42 ± 0.54).

Color changes in the bright red passion fruit pulp did not present significant differences among the treatments applied (*p* > 0.05), with an average ∆E of 3.48. Although the US-20 sample of yellow passion fruit pulp presented a ∆E value lower than 1.5, no significant difference was observed compared with the US-60 sample. It is important to highlight that, although the ∆E value exceeded 1.5 in some HIUS-processed samples in this study, the highest ∆E observed was 3.78, suggesting a color change that is minimally perceptible to the naked eye.

The preservation of the color of the bright red passion fruit pulp, estimated by the color difference parameter (ΔE), was also confirmed by the browning index (BI), in which all samples exhibited values statistically similar to those of the control sample (*p* > 0.05). On the other hand, the browning index indicated that prolonged thermal treatment (PS-63) resulted in a 40.60% reduction in the browning of yellow passion fruit pulp, which may be related to the increase in the L* color coordinate observed after the treatment.

Rodríguez-Rico et al. [49] evaluated pasteurized melon juice (65 °C/30 min) and HIUS-processed juice treated for 10 and 15 min under temperature control (10 ± 2 °C) and observed color differences only in the sonicated samples. The HIUS-treated juice samples exhibited ∆E values between 1.5 and 3.0, while the pasteurized juice presented a value of approximately 1.2. The authors associated the minimal color changes in HIUS-processed melon juice with the action of antioxidant compounds extracted during cavitation, a characteristic that may justify the minimal color changes observed in the HIUS-treated passion fruit pulps in this study (∆E between 1.15 and 3.78).

On the other hand, Ramirez-Corona et al. [50] evaluated orange juice processed by HIUS under controlled temperature (15 °C) for 20 min and observed perceptible color changes (∆E > 1.5), with the increase in the a* parameter being the most pronounced. According to the authors, these alterations were related to pigment oxidation reactions promoted by free radicals formed during HIUS processing. In the case of orange juice, the authors suggested that the carotenoids contributing to its yellow coloration were degraded.

Thus, these results indicate that HIUS processing may represent a promising alternative to conventional thermal treatment, as it is capable of more efficiently preserving the initial color and maintaining the visual freshness of the pulp. However, although instrumental color parameters provide objective indicators of quality, sensory evaluations are essential to determine whether these changes are perceptible and acceptable to consumers.

### 3.3. Cloud Stability and Centrifugal Sedimentation

Fruit pulp is a colloidal system composed of dispersed particles suspended in an aqueous phase. These suspended particles are primarily formed by polysaccharides such as cellulose and pectin, as well as plant cell tissue, cell fragments, and other insoluble materials. The homogeneity of fruit pulp is a relevant sensory attribute for the food industry, as it is strongly associated with product quality and consumer acceptance [51,52]. In this context, Table 2 presents the effect of HIUS on cloud stability and centrifugal sedimentation of passion fruit pulp (*Passiflora edulis* Sims.).

HIUS significantly increased the centrifugal sedimentation (*p* < 0.05) of both bright red and yellow passion fruit (*Passiflora edulis* Sims.) pulps compared with the control sample and the thermal treatment (PS-63). No statistically significant difference was observed between the control and the thermal treatment (PS-63) (*p* > 0.05), with mean values of 11.08 ± 0.14 and 10.96 ± 0.34 for the bright red and yellow passion fruit pulps, respectively. Furthermore, it was observed that centrifugal sedimentation in bright red passion fruit pulp was lower after the US-60 treatment compared with the US-20 treatment (21.44% and 28.38%, respectively). Conversely, the percentage of centrifuged sediments in yellow passion fruit pulp was significantly higher after the US-60 treatment compared with US-20 (29.60% and 19.00%, respectively).

Although the shear forces generated by HIUS are often associated with reducing the size of fibrous particles in pulp and decreasing the percentage of centrifugal sedimentation, a distinct effect was observed in this study. One factor that may explain the results obtained for passion fruit pulp is the HIUS processing parameters employed, which likely increased the release of intracellular material and consequently raised the number of small dispersed particles, resulting in a product with a more viscous appearance [53].

Rojas et al. [53] evaluated the effect of HIUS (793.65 W/cm^2^) under temperature control (22 ± 3 °C) in peach juice and reported that shorter processing times (3 min) promoted an increase in juice sedimentation. Additionally, the authors observed that samples processed for longer durations (6, 10, and 15 min) exhibited no sedimentation. Similarly, in a study by Rodríguez-Rico et al. [49], the application of HIUS (27 and 52 W/cm^2^) for longer periods (10 and 15 min) reduced melon juice sedimentation by up to 2.4%.

To determine the stability of suspended particles in passion fruit pulp, cloud stability analysis was performed (Table 2). This analytical method establishes that the centrifugation speed (4200× *g*) and time used (15 min) can indicate product stability for up to one year of storage. Thus, higher cloud stability values demonstrate that suspended particles in the pulp will remain uniformly dispersed over time [54]. In this study, the control sample of bright red passion fruit pulp exhibited a cloud stability of 11.70%, a characteristic that was maintained after the US-20 and PS-63 treatments (mean 11.53%) (*p* > 0.05). Conversely, a decrease in cloud stability was observed in the US-60 sample (6.20%). In yellow passion fruit pulp, cloud stability decreased after both US-60 and US-20 treatments (24.07% and 13.08%, respectively), an effect that may be related to the increase in suspended particles as previously discussed.

Overall, the cloud stability values observed in both treated and untreated samples were low (<35%), suggesting that suspended particles in passion fruit pulp (*Passiflora edulis* Sims.) are prone to sedimentation during storage. This effect may be associated with intrinsic characteristics of passion fruit pulp, such as the potential accumulation of large particles that create density differences relative to the aqueous phase, contributing to less stable suspensions [55]. Consequently, the HIUS parameters applied, such as processing time and power, may have been insufficient to improve the stability characteristics of passion fruit pulp [25].

Manzoor et al. [56] evaluated the effect of thermosonication at 200, 400, and 600 W for 20 min on spinach juice and reported that HIUS was effective in increasing cloud stability by approximately 10% at all studied power levels. Xing et al. [25] investigated the effect of HIUS at 810 W for 20 min on strawberry pulp, where cloud stability in the control sample was 50.38%, and observed a 17.31% increase after processing. The authors suggested that this increase may be related to the reduction in pulp particle size following cavitation.

### 3.4. Rheological Properties and Apparent Viscosity

The rheological behavior of bright red passion fruit pulp and yellow passion fruit pulp (*Passiflora edulis* Sims.) is presented in Table 3.

The consistency index (K) reflects the resistance of the fluid to the applied shear flow; that is, the higher the K value, the greater the viscosity of the material. In this regard, the US-60 treatment significantly increased the consistency index of the passion fruit pulps evaluated in this study. Conversely, the K value decreased after the US-20 treatment. The flow behavior index (n) was lower than 1.0 for all treatments, confirming that the fluids exhibit pseudoplastic behavior, a common characteristic in foods. The n value was significantly higher in all treated samples compared with the control sample (*p* < 0.05), indicating an increase in the pseudoplasticity of the passion fruit pulps. Furthermore, the results of this study suggest that the increase in viscosity was not influenced solely by temperature. The apparent viscosity of the pulps, determined at a shear rate of 100 s^–1^, showed an average increase of approximately 3.05% after the US-60 treatment, resulting in the highest apparent viscosity value among the treatments evaluated.

**Figure 2:**
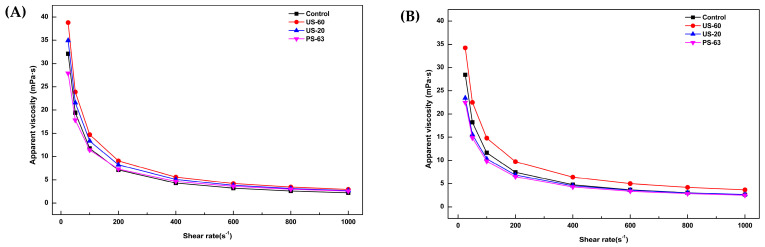
Effect of HIUS processing and thermal treatment on: (**A**) Apparent viscosity of bright red passion fruit pulp; (**B**) Apparent viscosity of yellow passion fruit pulp.

Flow curves of bright red and yellow passion fruit pulp for the different treatments are shown in Figure 2A,B.

The apparent viscosity of all samples decreased as the shear rate increased, demonstrating non-Newtonian fluid characteristics. This analysis also showed that bright red passion fruit pulp is naturally more viscous than yellow passion fruit pulp. After the treatments, the US-60 sample of bright red passion fruit pulp exhibited higher apparent viscosity compared with the other treatments, particularly at shear rates between 100 and 200 mPa·s. However, in yellow passion fruit pulp, the US-60 sample showed significantly higher viscosity at all evaluated shear rates (25 to 1000 mPa·s).

Overall, from the shear rate of 400 mPa·s onward, the control, US-20, and PS-63 samples presented similar apparent viscosity. These results indicate that the US-60 treatment increased the apparent viscosity of passion fruit pulp, especially in yellow passion fruit pulp. The energy released during HIUS processing promotes the rupture of cellular structures and the breakdown of polymeric chains, contributing to the increase in soluble solids content in the pulp. In addition, the reduction in particle size and modification of particle distribution may increase the total surface area, which may explain the increased apparent viscosity observed in the HIUS-processed pulp (US-60) [47].

Similar results were reported by Wang et al. [47], when evaluating the rheological properties of kiwi juice treated with HIUS at 400 W (20 kHz). The authors observed higher apparent viscosity in samples treated for 4, 12, and 16 min and attributed this behavior to structural modifications occurring in the juice during HIUS processing.

Chen et al. [57], when evaluating the rheological properties of strawberry pulp at shear rates between 10 and 200 s^–1^, reported a 397% increase in the K value in pulp treated with HIUS at 605 W/cm^2^ at 45 °C for 16 min, along with improved n values, indicating decreased pseudoplasticity.

### 3.5. Ascorbic Acid Retention Profile and Residual Activity of Ascorbate Oxidase

**Figure 3:**
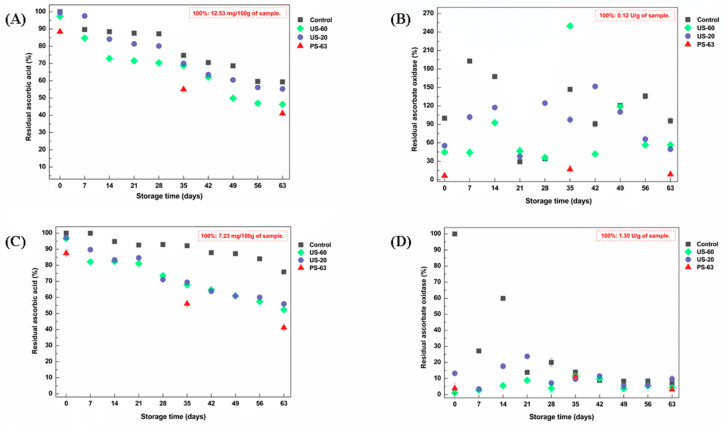
Effect of HIUS processing and thermal treatment on the residual ascorbic acid content and the residual activity of ascorbate oxidase (ASO) in *Passiflora edulis* Sims. pulps, respectively: (**A**,**B**): bright red passion fruit; (**C**,**D**): yellow passion fruit. Results are expressed as residual mean ± standard deviation. The red dashed line (- - -) represents the overall mean of the control sample at 0 days of storage.

Ascorbic acid (vitamin C) is an essential antioxidant for human health and can be easily degraded by exposure to oxygen, elevated temperatures, and/or the catalytic action of enzymes such as ascorbate oxidase (ASO) [58]. The retention of ascorbic acid and the residual activity of ASO during storage are illustrated in Figure 3.

A prominent reduction in ascorbic acid content was observed in bright red passion fruit pulp (*Passiflora edulis* Sims.) with increasing storage time at −18 °C (Figure 3A). Up to 7 days of storage, the pulp processed without heating (US-20) retained the highest ascorbic acid content (97.60%), which was 12.89% higher than that of the control sample at day 7. More pronounced degradation was observed after 14 days of storage in the US-60 sample (72.97%). However, after 35 days of storage, a marked resumption of degradation was evident in all evaluated conditions, with retention values of 74.75% (control), 68.81% (US-60), 70.03% (US-20), and 55.03% (PS-63). After 63 days of storage, the US-20 sample retained 55.49% of ascorbic acid (a decrease of only 4.09% relative to the control sample at day 63).

Limited enzymatic inactivation was observed in bright red passion fruit pulp (Figure 3B). ASO activity increased between days 7 and 14 of storage, especially in the control sample (increases of 192.82% and 167.64%, respectively). Reflecting the ascorbic acid degradation profile, after 35 days of storage there was a marked resurgence in ASO activity, which increased by 146.85% in the control sample, 249.90% in the US-60 sample, and 97.58% in the US-20 sample. After 63 days of storage, ASO activity remained below 60% in the HIUS-treated samples (US-60 and US-20).

A significant reduction in ascorbic acid content was also observed in yellow passion fruit pulp (*Passiflora edulis* Sims.) during storage (Figure 3C). More pronounced degradation occurred after the seventh and twenty-eighth days of storage in the samples treated with HIUS and thermal processing. After 28 days, ascorbic acid content ranged from 73.61% (US-60) to 71.07% (US-20), representing 19.28% and 21.82% lower values than those of the control sample at the same timepoint, respectively. The degradation pattern in yellow passion fruit pulp remained similar up to 63 days of storage, remaining above 50% in the HIUS-treated samples (US-60 and US-20).

As shown in Figure 3D, ASO activity was pronounced in yellow passion fruit pulp (1.30 U/g); however, it showed little relationship with ascorbic acid degradation. For this sample, residual enzyme activity ranged from 7.19% to 59.90% (control), 1.15% to 11.73% (US-60), 3.54% to 23.83% (US-20), and 3.20% to 11.24% (PS-63) between day 0 and day 63 of storage.

Overall, based on the data for both bright red and yellow passion fruit pulps, the HIUS treatments US-60 and US-20 were more effective in preserving ascorbic acid compared with thermal treatment (PS-63), while ASO was only partially inactivated by HIUS and partially regained activity during storage. However, the findings of this study suggest that ASO activity exerted limited influence on the ascorbic acid degradation profile in passion fruit pulp. The instability of ASO observed in this study resembles the trends reported by Fonteles et al. [59] when applying HIUS at power levels above 226 W/cm^2^ to cantaloupe melon juice, where ASO was found to be more unstable than other evaluated peroxidases. The authors also reported a decrease in ASO activity after HIUS processing, while the same power applied for 6 min promoted an increase in enzymatic activity.

The results of this study demonstrated that thermal treatment (PS-63) resulted in lower ASO activity in the evaluated pulps, confirming the efficiency of thermal processing in enzyme inactivation. However, lower preservation of ascorbic acid was observed in samples subjected to thermal treatment at 63 °C for 30 min (PS-63). This reduction can be attributed to the high sensitivity of this compound to thermal degradation and oxidation induced by heating. A similar outcome was reported by Ordóñez-Santos et al. [60], who observed more pronounced ascorbic acid degradation in Cape gooseberry juice pasteurized at 80 °C for 10 min compared with HIUS-treated samples.

Although HIUS does not use heat as its primary mechanism of action, acoustic cavitation can promote alterations in the secondary and tertiary structures of proteins, leading to enzyme inactivation [16]. However, moderate processing power can also increase enzymatic activity. Additionally, the free radicals generated during bubble expansion and collapse in the liquid during HIUS processing can contribute to enzyme inactivation [15,60].

The reduction in ascorbic acid levels observed during storage in the HIUS-treated samples compared with control samples may be attributed to the acoustic cavitation phenomenon generated during treatment. This effect promotes the formation and collapse of microbubbles, enhancing the incorporation of molecular oxygen into the food matrix. Consequently, greater interaction occurs between oxygen and bioactive compounds, resulting in ascorbic acid oxidation and its degradation during storage. L-ascorbic acid, which represents the active form of ascorbic acid, is converted during oxidative processes into L-dehydroascorbic acid, which is then rapidly hydrolyzed to 2,3-diketo-L-gulonic acid, ultimately leading to the loss of biological activity [60,61]. A second factor that may have contributed to the reduction in this vitamin during storage is its interaction with free radicals (H_2_O → H· + ·OH) formed during water sonolysis [62].

Thus, these results reveal that HIUS processing may represent a promising alternative to conventional thermal treatment, capable of partially inactivating ASO while simultaneously promoting the retention of ascorbic acid in passion fruit pulp. This effect reinforces the potential of HIUS processing, which aligns with growing consumer demand for minimally processed products with enhanced nutritional quality.

### 3.6. Total Carotenoids

Carotenoids are a class of liposoluble pigments responsible for the yellow, orange, and red coloration of fruits and other photoautotrophic organisms [63]. In this study, it was observed (Table 4) that the treatments preserved the total carotenoids (TC) in bright red and yellow passion fruit pulps (*Passiflora edulis* Sims.).

The mean carotenoid contents expressed as β-carotene were 16.35 ± 0.63 and 13.73 ± 0.74 β-carotene µg g^−1^, respectively, whereas the carotenoid levels expressed as lycopene were substantially lower (9.02 ± 0.22 and 6.93 ± 0.32 lycopene µg g^−1^, respectively).

The yellow–orange coloration of *Passiflora edulis* Sims. pulp results from carotenoid biosynthesis during physiological fruit development, particularly β-carotene [64]. Typically, these compounds are stored inside plant cells through physical and chemical associations. During HIUS processing, pressure fluctuations generated by shear forces may promote the disruption of cellular bonds and the release of compounds [47].

Based on these considerations, one hypothesis that may explain the preservation of carotenoids in *Passiflora edulis* Sims. pulp is the high stability of intracellular bonds and carotenoid structures under the energy intensity applied during processing. Ordóñez-Santos et al. [60] reported that carotenoid stability may also be associated with the degradation of other antioxidants, such as ascorbic acid, which reacts with free radicals formed during HIUS processing.

These findings support the idea that HIUS processing can preserve the chemical compounds responsible for the color of fruit pulp, such as carotenoids. In this context, this technique may represent an interesting alternative for industrial passion fruit pulp processing, as this parameter is directly linked to consumer perception of overall product quality [65].

### 3.7. Total Phenolic Content and Antioxidant Capacity

Phenolic compounds are molecules derived from the specialized metabolism of plants and are responsible for numerous biological activities due to their antioxidant potential [66,67]. The total phenolic content (TPC) and antioxidant activity are presented in Table 4. In this study, it was observed that the application of the US-60 treatment increased the TPC of bright red passion fruit pulp (*Passiflora edulis* Sims.) (a 10.13% increase compared to the control sample). In addition, the US-20 and PS-63 treatments significantly increased TPC values. However, no significant changes in TPC were observed for yellow passion fruit pulp (*Passiflora edulis* Sims.) after the treatments, and the overall mean was 42.59 ± 0.46.

The increased TPC may be explained by the formation of pores in the plant cell membrane after HIUS treatment, which promotes increased mass transfer. Furthermore, shear waves may induce the disruption of intracellular bonds between phenolic compounds, resulting in a higher extracellular phenolic content in the HIUS-treated sample [22].

Wang et al. [68] analyzed strawberry juice treated by HIUS for 12 min at 400 W and reported that the phenolic content increased significantly. A similar result was found by De Araújo et al. [15] when evaluating araçá-boi pulp treated by HIUS for 8.75 min at a power of 400 W, where they observed a 13.53% increase compared to the untreated pulp.

Despite the increased TPC reported in the US-60 sample of bright red passion fruit pulp, this effect did not translate into enhanced antioxidant capacity in the DPPH and ABTS assays, in which the mean values for the treatments showed no significant differences compared to the control sample (mean values of 1.59 and 3.73 µM TE g^−1^ for DPPH and ABTS, respectively).

Antioxidant capacity also did not differ significantly in yellow passion fruit pulp based on the DPPH and ABTS assays, with mean values of 1.12 and 3.14 µM TE g^−1^, respectively. Although studies demonstrate that the antioxidant capacity of *Passiflora* species pulp is strongly associated with phenolic compounds and the chemical structures of phenolic molecules, a possible explanation for the increased TPC not influencing the antioxidant activity of bright red passion fruit pulp is that the predominant antioxidant contribution may be associated with other compounds, such as carotenoids [69,70,71].

Additionally, studies report that increases in bioactive compound content, and consequently in antioxidant capacity, are strongly related to the duration and power of HIUS processing. This effect was observed by Oliveira et al. [22], who reported an increase in TPC (10.94% compared to the control) and maintenance of antioxidant activity in açaí juice after increasing HIUS processing power to 700 J·mL^−1^.

### 3.8. Phenolic Compound Profile

The phenolic compound profile of bright red and yellow passion fruit pulp (*Passiflora edulis* Sims.) was significantly influenced by HIUS and thermal treatment, as reported in Table 5.

Comparison of the mean values of quantified phenolic compounds revealed dynamic variations that reflected the influence of treatments on the release or degradation of these compounds. In bright red passion fruit pulp, catechin content increased markedly after treatment. The control sample exhibited 189.45 μg 100 g^−1^ DW, which increased by 173.96% after US-60 treatment (519.01 μg 100 g^−1^ DW). An increase in catechin content was also observed after HIUS at 20 ± 2 °C (US-20) and after thermal treatment (PS-63), with rises of 7.25% and 15.87%, respectively, compared to the control. A similar trend was observed in yellow passion fruit pulp, with increases of 5.90% and 6.84% in catechin content following US-60 and US-20 treatments, respectively. Conversely, thermal treatment at 63 °C for 30 min (PS-63) reduced catechin content in yellow passion fruit pulp to 379.35 μg 100 g^−1^ DW, an 11.55% decrease relative to the control.

These increases may be attributed to the disruption of intracellular bonds involving catechin promoted by mechanical action and by the generation of free radicals resulting from acoustic cavitation, both induced by HIUS, which facilitate catechin extraction into the extracellular medium [22].

Another possible explanation for the increased catechin content is that HIUS processing may generate additional hydroxyl groups that can interact with the aromatic ring of the molecule, favoring the biosynthesis of phenolic compounds in the pulp [68]. Furthermore, the results indicate that HIUS treatment combined with mild heating (final processing temperature of 59.5 °C) was significantly more effective in enhancing catechin levels in bright red passion fruit pulp. In contrast, the reduction observed in yellow passion fruit pulp subjected to thermal treatment (PS-63) may be explained by oxidative and thermal hydrolysis processes that likely contributed to catechin degradation.

Phenolic acids exhibited mixed responses to the treatments in both pulps. Vanillic acid increased by 4.60% in bright red passion fruit pulp after US-20 treatment. Similarly, vanillic acid levels increased by an average of 5.89% in yellow passion fruit pulp following US-60 and US-20 treatments. This increase may be associated with chemical and enzymatic hydrolysis reactions induced by HIUS processing, which likely promoted the release of phenolic acids, such as vanillic acid, previously bound to other chemical components, such as glycosides, or to structures within the cell–matrix [15,72].

Regarding gallic acid levels, stability was observed across treatments in yellow passion fruit pulp, with a mean of 3.24 μg 100 g^−1^ DW, whereas gallic acid was not detected in bright red passion fruit pulp. Syringic acid levels also remained stable in both bright red and yellow passion fruit pulps after US-60 treatment, averaging 143.66 and 45.43 μg 100 g^−1^ DW, respectively. However, syringic acid content decreased from 139.35 μg 100 g^−1^ DW to 119.05 μg 100 g^−1^ DW after thermal treatment (PS-63) in bright red passion fruit pulp. Similarly, syringic acid levels in yellow passion fruit pulp decreased from 48.14 μg 100 g^−1^ DW in the control sample to 13.42 μg 100 g^−1^ DW after thermal treatment (PS-63), representing a 72.12% reduction. The decline in syringic acid levels after thermal treatment is related to the possible thermal degradation of this compound under more severe processing conditions [16].

Some phenolic acids, such as caffeic and chlorogenic acids, showed similar patterns in bright red passion fruit pulp. Caffeic acid decreased moderately from 30.46 μg 100 g^−1^ DW in the control to an average of 24.50 μg 100 g^−1^ DW after US-60 and US-20 HIUS treatments (an average reduction of 19.58%). Chlorogenic acid levels also decreased after both HIUS and thermal treatments. Notably, US-60 markedly reduced chlorogenic acid content from 77.03 μg 100 g^−1^ DW in the control to 10.42 μg 100 g^−1^ DW, representing an 86.47% reduction. The reductions in caffeic and chlorogenic acids may be related to oxidative degradation promoted by HIUS processing. The energy delivered during HIUS may stimulate chemical reactions leading to alterations in polyphenol chemical structures as well as degradation into smaller molecular fragments [16]. In yellow passion fruit pulp, caffeic acid remained stable across treatments, with a mean of 9.63 μg 100 g^−1^ DW. Chlorogenic acid decreased from 29.78 μg 100 g^−1^ DW in the control to 25.33 μg 100 g^−1^ DW after thermal treatment at 63 °C for 30 min (PS-63), reflecting a 14.94% reduction.

Ferulic acid levels remained stable in yellow passion fruit pulp after US-60 and US-20 treatments, with a mean of 104.43 μg 100 g^−1^ DW. However, an 8.24% decrease occurred after prolonged thermal treatment (PS-63). Ferulic acid was not detected in bright red passion fruit pulp. Sinapic acid in bright red passion fruit pulp remained stable after US-60 and PS-63 treatments (average of 1.60 μg 100 g^−1^ DW) but was not detected after US-20. Similarly, in yellow passion fruit pulp, sinapic acid was stable under US-60 treatment, with an average of 2.78 μg 100 g^−1^ DW. However, its levels decreased from 2.56 μg 100 g^−1^ DW in the control to 0.98 μg 100 g^−1^ DW after US-20 treatment (a 61.72% reduction).

Based on these results, it is possible to infer that HIUS may be effective for processing passion fruit pulp, promoting the increase or preservation of phenolic compounds under at least one of the HIUS conditions tested. Ismail et al. [73] evaluated the effect of different HIUS intensities on the phenolic compounds of baobab pulp and compared them with thermal treatment. The authors reported that all HIUS-treated samples exhibited significantly higher phenolic content than samples subjected to thermal treatment.

Similarly, Yıkmıs et al. [24] evaluated the effect of HIUS with and without mild heating on the phenolic compounds of broccoli juice and reported that both HIUS conditions were effective in maintaining, and in some cases increasing, phenolic compound levels. However, thermal treatment reduced the content of specific compounds, such as hydroxybenzoic and protocatechuic acids. The authors emphasized that processing conditions can directly influence the stability of phenolic compounds.

Overall, the findings of this study suggest that HIUS processing for 5 min combined with mild heating (US-60) may be a promising approach to balance the release and preservation of the phenolic compounds evaluated, compared with the other treatments. These results support the hypothesis that optimized HIUS processing conditions may be an effective strategy for enhancing the phenolic profile of passion fruit pulp.

### 3.9. Microorganism Count

The assessment of natural microbiological contamination by mesophilic and aerobic bacteria, molds and yeasts, total and thermotolerant coliforms, *Escherichia coli*, and *Salmonella* spp. in the passion fruit pulps is presented in Table 6.

The treatments did not show a significant effect on the mesophilic and aerobic bacterial counts of yellow passion fruit (*Passiflora edulis* Sims.) pulp, with an average value of 1.77 log CFU/mL. The use of HIUS significantly reduced mold and yeast counts in the evaluated passion fruit pulps, and the results obtained were below the limit established by Brazilian regulations (maximum of 3.3 log CFU/mL) [74].

Some microorganisms, such as mesophilic and aerobic bacteria, as well as molds and yeasts, are widely distributed across different environments and are common components of the food microbiome. Typically, when present at low counts, these microorganisms do not pose significant risks to human health [16]. HIUS also yielded satisfactory results in the inactivation of total and thermotolerant coliforms identified in the yellow passion fruit (*Passiflora edulis* Sims.) pulp. The detection of total and thermotolerant coliforms in the control sample of yellow passion fruit (*Passiflora edulis* Sims.) may be associated with extrinsic factors such as the presence of environmental microorganisms and non-sterile handling and/or storage conditions [75]. However, no growth of colonies suspected as *Escherichia coli* (MPN/mL) was detected in samples subjected to biochemical testing. Regarding the detection of *Salmonella* spp., absence was confirmed in all samples evaluated.

The naturally acidic pH of passion fruit pulp may slow down or inhibit the growth of pathogenic bacteria [26,76]. However, the reduction in microorganisms in the passion fruit pulps after HIUS treatment may be attributed to the physical, chemical, and mechanical effects occurring during cavitation [77]. During cavitation, the collapse of microbubbles can interact with the hydrophobic surface of microorganisms, causing severe damage to the cell wall and consequent microbial inactivation. Moreover, more rigid microbial cellular structures can be inactivated by free radicals generated during the sonolysis of water molecules, leading to exposure and alterations of microbial DNA [26].

HIUS processing conditions applied for 5 min, with and without heating (US-60 and US-20, respectively), demonstrated satisfactory performance, particularly in reducing mold and yeast counts in the analyzed passion fruit pulps. These microorganisms are responsible for food spoilage, promoting undesirable sensory changes such as off-odors and unpleasant textures. Furthermore, some fungal species may synthesize mycotoxins that are potentially harmful to human health [78]. These results suggest that HIUS processing may be an effective technique for promoting microbiological inactivation without significantly compromising the nutritional quality of the product. However, future studies should include investigations that evaluate microbial counts over time in order to determine the microbiological quality throughout the product’s shelf life.

### 3.10. Principal Component Analysis

**Figure 4:**
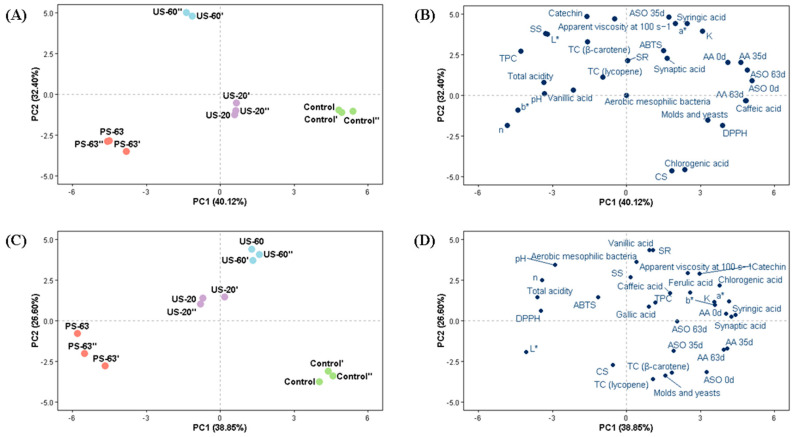
Principal component analysis (PCA). (**A**) Score plot and (**B**) loading plot of bright red passion fruit pulp. (**C**) Score plot and (**D**) loading plot of yellow passion fruit pulp.

Principal component analysis was used to evaluate the effects of HIUS and thermal treatment on the passion fruit pulps (Figure 4). Figure 4A,B show the PCA results for bright red passion fruit pulp. The first two dimensions accounted for 40.12% and 32.40% of the total variance, respectively, explaining 72.52% of the data. This value indicates that PCA captured a substantial portion of the information contained in the dataset.

Figure 4A illustrates the PCA results, in which the different treatments were grouped according to their scores in the two principal dimensions. On the positive side of PC1, the control and US-20 variables were clustered, indicating minimal influence of US-20 on the evaluated characteristics of the pulp, due to its similarity to the control sample. Notably, PC1 differentiated the control and US-20 samples from the PS-63 sample, confirming that thermal treatment negatively affected the initial quality parameters of the pulp. PC2 showed a clear distinction between the US-60 and PS-63 treatments. The US-60 treatment was positioned on the positive side of PC2, while PS-63 was grouped on the negative side, indicating opposite behaviors between the treatments. Furthermore, the positional difference between US-60 and US-20 suggests that mild heating during HIUS processing may significantly influence the parameters evaluated in bright red passion fruit pulp.

Figure 4B shows the contribution of variables to the PCA in bright red passion fruit pulp. From these results, it is possible to observe that the variables were distributed mainly in the upper portion of the plot. The US-60 condition was primarily characterized by higher values of soluble solids (SS), total acidity, lightness (L*), apparent viscosity at 100 s^−1^, centrifuged sediments (SR), total carotenoids (expressed as β-carotene and lycopene), TPC, catechin, and vanillic acid, indicating that this treatment may be promising for preserving and increasing the content of bioactive compounds, in addition to promoting significant physical and chemical changes in bright red passion fruit pulp.

Notably, the control and US-20 samples exhibited higher levels of cloud stability (CS), DPPH, caffeic acid, chlorogenic acid, and ascorbic acid after 63 days of storage, indicating that US-20 is effective in preserving physical stability, bioactive compounds, and antioxidant activity in bright red passion fruit pulp. However, HIUS processing in the absence of heating (US-20) also resulted in high mold and yeast counts, suggesting that this treatment had a lower capacity to inactivate spoilage microorganisms. Thermal treatment at 63 °C for 30 min (PS-63) showed higher values of pH, the b* color coordinate, and n, indicating a more acidic, more yellowish pulp with characteristics of a less pseudoplastic fluid. The remaining variables were clustered on the positive sides of PC1 and PC2, suggesting a strong association of these components with the control sample and the HIUS-treated samples. This behavior indicates a positive influence of HIUS treatment, particularly in preserving and enhancing the bioactive compounds of bright red passion fruit pulp. On the other hand, it is important to highlight that the sample subjected to thermal treatment was clearly differentiated, showing significant distinctions in quality attributes compared with the samples treated with HIUS (US-60 and US-20).

Figure 4C,D illustrate the PCA results for yellow passion fruit pulp. The first two dimensions explained 38.85% and 26.60% of the variance, respectively, and together accounted for a substantial portion of the information in the dataset. Figure 4C shows that PC1 grouped the control and US-60 variables, highlighting a positive correlation between US-60 and the analyses located on the positive side of PC1. Similar to the results observed for bright red passion fruit pulp, PC1 also distinctly separated the control sample from the thermally treated sample (PS-63), revealing that thermal treatment negatively influenced the initial quality parameters of the pulp. PC2 revealed a subtle difference between the US-20 and US-60 samples, suggesting that mild heating influenced the quality characteristics of yellow passion fruit pulp.

Figure 4D presents the contribution of variables to the PCA for yellow passion fruit pulp, where it is possible to observe that the variables were concentrated on the right side of the plot, a region associated with the control sample and the US-60 sample. The control sample exhibited higher values of total carotenoids (expressed as β-carotene and lycopene), AA at 35 and 63 days, ASO activity at 35 days, and mold and yeast counts. These results indicate that HIUS and thermal treatments are associated with reductions in microbial counts and enzymatic activity during storage, mechanisms related to the degradation process of yellow passion fruit pulp, although they promote substantial losses in ascorbic acid content during storage. The US-60 sample was characterized by higher values of AA at 0 days, TPC, catechin, ferulic acid, syringic acid, and the other phenolics evaluated, indicating that this treatment was effective in preserving and enhancing bioactive compounds present in yellow passion fruit pulp. In addition, the US-60 treatment showed high values of the color parameters a* and b*, consistency index (K), apparent viscosity at 100 s^−1^, and centrifuged sediments (SR).

These results suggest enhancement of the orange hue of yellow passion fruit pulp, increased viscosity, and a higher percentage of centrifuged sediments, indicating a greater amount of suspended particles compared with the other treatments. The US-20 treatment exhibited higher antioxidant activity (DPPH and ABTS), lower pseudoplasticity, and higher pH and total acidity values. Conversely, the thermal treatment (PS-63) grouped only two variables, showing higher values for the L* color coordinate and greater cloud stability (CS), indicating a lighter and more stable sample during storage.

## 4. Conclusions

This study demonstrated that high-intensity ultrasound (HIUS) is a promising non-thermal technique for processing bright red and yellow passion fruit pulps (*Passiflora edulis* Sims.), promoting partial inactivation of ascorbate oxidase (ASO) and effectively reducing spoilage and pathogenic microorganisms, such as molds, yeasts, and total and thermotolerant coliforms. In addition, HIUS enhanced the catechin content and preserved the levels of vanillic acid, syringic acid, and ferulic acid in the passion fruit pulps. The use of HIUS combined with mild heating (US-60) provided an optimal balance between improving the phenolic compound profile and ensuring enzymatic and microbiological inactivation in the evaluated pulps. However, the application of HIUS in the absence of heating also produced promising results regarding the preservation of the initial quality characteristics of the pulp. Compared with conventional thermal treatment, HIUS processing, with or without mild heating, demonstrated superior retention of bioactive compounds.

HIUS treatment alone increased suspended particles and viscosity characteristics of the passion fruit pulp. Therefore, future studies should evaluate its combination with sustainable clarification techniques, such as enzymatic treatment, centrifugation, and membrane filtration. In an industrial context, HIUS processing presents scalability potential, as it can be integrated into continuous production lines, reducing pulp processing time and offering lower energy consumption compared with conventional techniques. In addition to contributing to products with improved quality attributes, the use of HIUS eliminates the need for synthetic antioxidants and antimicrobial agents, demonstrating its promise as a sustainable tool for producing pulps with longer shelf life and clean-label characteristics. Future research should investigate sensory perception, industrial and economic feasibility, optimal storage conditions (packaging, freezing and refrigeration), shelf-life behavior, and standardized processing parameters to maximize the functional benefits of this technology.

## Figures and Tables

**Table 1:** Parameters of the treatments applied to *Passiflora edulis* Sims. pulp.

Varieties	Code/ Treatments	Power (Watts)	Energy Density (J/cm^3^)	Time (min)	Initial Temperature (°C)	Final Temperature (°C)
**Bright red passion fruit** (*Passiflora edulis* Sims.)	Control	-	-	-	-	-
	PS-63	-	-	30	22.0	63.0
	US-60	40	211.12	5	24.0	59.5
	US-20	40	228.87	5	20.3	23.3
**Yellow** **passion fruit** (*Passiflora edulis* Sims.)	Control	-	-	-	-	-
	PS-63	-	-	30	21.0	63.0
	US-60	40	212.37	5	22.8	59.17
	US-20	40	227.68	5	20.3	23.0

**Table 2:** Effect of HIUS and pasteurization on the pH, soluble solids, total acidity, color, centrifugal sedimentation, and cloud stability of bright red and yellow passion fruit pulp (*Passiflora edulis* Sims.).

Parameters	Treatments			
	Control	US-60	US-20	PS-63
**Bright red passion fruit**				
pH	2.66 ± 0.02 ^b^	2.71 ± 0.01 ^a^	2.73 ± 0.01 ^a^	2.71 ± 0.01 ^a^
Soluble solids (°Brix)	14.00 ± 0.10 ^c^	14.77 ± 0.06 ^a^	14.20 ± 0.10 ^bc^	14.37 ± 0.15 ^b^
Total acidity (%)	3.95 ± 0.11 ^a^	4.06 ± 0.01 ^a^	3.93 ± 0.06 ^a^	4.09 ± 0.08 ^a^
L*	45.10 ± 0.08 ^c^	48.33 ± 0.57 ^a^	46.88 ± 0.10 ^b^	46.58 ± 0.01 ^b^
a*	20.25 ± 0.42 ^ab^	20.86 ± 0.28 ^a^	20.03 ± 0.44 ^ab^	19.29 ± 0.03 ^b^
b*	26.50 ± 0.69 ^b^	28.29 ± 1.15 ^ab^	29.05 ± 0.39 ^ab^	29.57 ± 0.04 ^a^
h*	51.70 ± 0.04 ^b^	51.37 ± 0.71 ^b^	53.63 ± 0.28 ^a^	55.17 ± 0.23 ^a^
C*	30.04 ± 0.14 ^ab^	28.49 ± 0.70 ^b^	30.38 ± 0.19 ^a^	22.00 ± 0.29 ^c^
∆E	-	3.78 ± 1.07 ^a^	3.13 ± 0.23 ^a^	3.53 ± 0.04 ^a^
Browning index (BI)	115.44 ± 4.01 ^a^	113.79 ± 3.53 ^a^	120.73 ± 2.75 ^a^	123.16 ± 0.10 ^a^
Centrifugal sedimentation (%)	11.59 ± 0.10 ^c^	21.44 ± 0.75 ^b^	28.38 ± 1.57 ^a^	10.57 ± 0.18 ^c^
Cloud stability (%)	11.70 ± 0.44 ^a^	6.20 ± 0.78 ^b^	12.01± 0.51 ^a^	10.90 ± 0.50 ^a^
**Yellow passion fruit**				
pH	2.44 ± 0.04 ^b^	2.79 ± 0.03 ^a^	2.77 ± 0.01 ^a^	2.75 ± 0.01 ^a^
Soluble solids (°Brix)	12.20 ± 0.10 ^bc^	12.77 ± 0.06 ^a^	12.03 ± 0.06 ^c^	12.33 ± 0.06 ^b^
Total acidity (%)	3.98 ± 0.11 ^a^	4.13 ± 0.08 ^a^	4.08 ± 0.02 ^a^	4.20 ± 0.00 ^a^
L*	45.22 ± 0.01 ^b^	44.48 ± 0.46 ^b^	45.79 ± 0.18 ^b^	51.69 ± 0.49 ^a^
a*	18.56 ± 0.01 ^a^	17.79 ± 0.16 ^b^	18.02 ± 0.01 ^ab^	12.57 ± 0.24 ^c^
b*	23.62 ± 0.17 ^ab^	22.26 ± 0.76 ^b^	24.46 ± 0.24 ^a^	18.06 ± 0.18 ^c^
h*	52.61 ± 0.13 ^b^	53.59 ± 0.75 ^b^	55.42 ± 0.22 ^a^	56.87 ± 0.07 ^a^
C*	33.35 ± 0.80 ^a^	35.14 ± 1.09 ^a^	35.28 ± 0.57 ^a^	35.30 ± 0.01 ^a^
∆E	-	1.74 ± 0.86 ^b^	1.15 ± 0.26 ^b^	10.42 ± 0.54 ^a^
Browning index (BI)	100.01 ± 0.70 ^ab^	95.24 ± 2.16 ^b^	101.12 ± 0.50 ^a^	59.41 ± 1.52 ^c^
Centrifugal sedimentation (%)	11.21 ± 0.97 ^c^	29.60 ± 1.34 ^a^	19.00 ± 0.73 ^b^	10.72 ± 0.48 ^c^
Cloud stability (%)	30.99 ± 0.24 ^b^	24.07 ± 0.29 ^c^	13.08 ± 0.49 ^d^	33.08 ± 0.47 ^a^

The results were expressed as mean ± standard deviation (n = 3). Superscript lowercase letters (a–d) in the same line indicate statistically significant differences among treatments according to Tukey’s test (*p* ≤ 0.05). (L*) lightness; (a*) scale ranging from green (−a) to red (+a); (b*) scale ranging from blue (−b) to yellow (+b); (C*) Chroma; (∆E) color difference after HIUS or thermal treatment.

**Table 3:** Effect of HIUS and pasteurization on the rheological properties and on the apparent viscosity at 100 s^−1^ (m∙Pa·s) of bright red and yellow passion fruit pulp (*Passiflora edulis* Sims.).

Parameters	Treatments			
	Control	US-60	US-20	PS-63
**Bright red passion fruit**				
k (mPa∙s)	330.48 ± 0.15 ^b^	359.38 ± 0.01 ^a^	325.63 ± 0.10 ^c^	220.49 ± 0.00 ^d^
n	0.28 ± 0.00 ^c^	0.30 ± 0.00 ^b^	0.31 ± 0.00 ^b^	0.36 ± 0.00 ^a^
R^2^	0.951	0.986	0.975	0.962
Apparent viscosity at 100 s^−1^ (mPa∙s)	11.75 ± 0.04 ^c^	14.71 ± 0.11 ^a^	13.32 ± 0.00 ^b^	11.41 ± 0.07 ^d^
**Yellow passion fruit**				
k (mPa∙s)	223.38 ± 0.91 ^b^	240.18 ± 0.04 ^a^	157.29 ± 1.38 ^c^	154.00 ± 1.37 ^c^
n	0.36 ± 0.00 ^d^	0.39 ± 0.00 ^c^	0.41 ± 0.00 ^a^	0.40 ± 0.00 ^b^
R^2^	0.984	0.968	0.967	0.984
Apparent viscosity at 100 s^−1^ (mPa∙s)	11.64 ± 0.02 ^b^	14.79 ± 0.03 ^a^	10.32 ± 0.04 ^c^	9.81 ± 0.01 ^d^

Results are expressed as mean ± standard deviation. Superscript lowercase letters (a–d) in the same line indicate statistical differences among treatments according to Tukey’s test (*p* ≤ 0.05).

**Table 4:** Effect of HIUS and pasteurization on total carotenoids, total phenolic compounds (TPC), and antioxidant activity of bright red and yellow passion fruit pulp (*Passiflora edulis* Sims.).

Parameters	Treatments			
	Control	US-60	US-20	PS-63
**Bright red passion fruit**				
Total carotenoids (*β*-carotene µg g^−1^)	15.94 ± 0.06 ^a^	17.19 ± 0.09 ^a^	16.07 ± 0.53 ^a^	16.21 ± 1.85 ^a^
Total carotenoids (lycopene µg g^−1^)	9.02 ± 0.13 ^a^	9.13 ± 0.23 ^a^	8.84 ± 0.52 ^a^	9.09 ± 0.00 ^a^
TPC (mg GAE 100 g^−1^)	44.42 ± 0.01 ^c^	48.92 ± 0.16 ^a^	46.04 ± 0.67 ^b^	47.71 ± 0.19 ^a^
DPPH (µM TE g^−1^)	1.66 ± 0.04 ^ab^	1.51 ± 0.02 ^b^	1.67 ± 0.06 ^a^	1.51 ± 0.01 ^ab^
ABTS (µM TE g^−1^)	3.76 ± 0.00 ^a^	3.79 ± 0.20 ^a^	3.71 ± 0.18 ^a^	3.65 ± 0.07 ^a^
**Yellow passion fruit**				
Total carotenoids (*β*-carotene µg g^−1^)	15.02 ± 0.11 ^a^	13.25 ± 1.10 ^a^	12.91 ± 0.93 ^a^	13.73 ± 0.82 ^a^
Total carotenoids (lycopene µg g^−1^)	7.44 ± 0.18 ^a^	6.41 ± 0.57 ^a^	6.93 ± 0.42 ^a^	6.93 ± 0.12 ^a^
TPC (mg GAE 100 g^−1^)	42.74 ± 0.36 ^ab^	43.64 ± 0.73 ^a^	41.43 ± 0.70 ^b^	42.54 ± 0.06 ^ab^
DPPH (µM TE g^−1^)	1.05 ± 0.07 ^a^	1.09 ± 0.06 ^a^	1.17 ± 0.09 ^a^	1.18 ± 0.00 ^a^
ABTS (µM TE g^−1^)	3.10 ± 0.03 ^a^	3.14 ± 0.06 ^a^	3.19 ± 0.16 ^a^	3.14 ± 0.18 ^a^

Total phenolic content (TPC). GAE 100 g^−1^: gallic acid equivalents per 100 g. TE g^−1^: Trolox equivalents per gram. Results are expressed as mean ± standard deviation (n = 3). Superscript lowercase letters (a–c) in the same line indicate statistically significant differences among treatments according to Tukey’s test (*p* ≤ 0.05).

**Table 5:** Influence of HIUS and pasteurization on the phenolic compound profile of bright red and yellow passion fruit pulp (*Passiflora edulis* Sims.).

Phenolic Compound	Treatments			
	Control	US-60	US-20	PS-63
**Bright red passion fruit**				
*Flavonoids*				
Catechin	189.45 ± 0.02 ^c^	519.01 ± 0.10 ^a^	203.19 ± 0.11 ^bc^	219.51 ± 0.04 ^b^
Vanillic acid	202.50 ± 0.04 ^b^	207.58 ± 0.00 ^ab^	211.82 ± 0.02 ^a^	207.14 ± 0.03 ^ab^
Gallic acid	n.d.	n.d.	n.d.	n.d.
Syringic acid	139.35 ± 0.04 ^ab^	147.96 ± 0.01 ^a^	131.42 ± 0.02 ^b^	119.05 ± 0.02 ^c^
Caffeic acid	30.46 ± 0.00 ^a^	24.37 ± 0.00 ^b^	24.62 ± 0.01 ^b^	22.99 ± 0.00 ^c^
Chlorogenic acid	77.03 ± 0.00 ^a^	10.42 ± 0.01 ^c^	66.01 ± 0.03 ^b^	60.10 ± 0.01 ^b^
Ferulic acid	n.d.	n.d.	n.d.	n.d.
Synaptic acid	2.02 ± 0.00 ^a^	1.90 ± 0.01 ^a^	n.d.	0.88 ± 0.01 ^a^
**Yellow passion fruit**				
*Flavonoids*				
Catechin	428.91 ± 0.11 ^b^	454.21 ± 0.09 ^a^	458.25 ± 0.08 ^a^	379.35 ± 0.05 ^c^
Vanillic acid	158.97 ± 0.00 ^b^	169.79 ± 0.02 ^a^	166.87 ± 0.02 ^a^	156.79 ± 0.03 ^b^
Gallic acid	3.21 ± 0.01 ^a^	3.54 ± 0.02 ^a^	3.33 ± 0.01 ^a^	2.86 ± 0.00 ^a^
Syringic acid	48.14 ± 0.02 ^a^	41.72 ± 0.02 ^a^	26.02 ± 0.02 ^b^	13.42 ± 0.03 ^c^
Caffeic acid	9.76 ± 0.00 ^a^	10.23 ± 0.00 ^a^	9.22 ± 0.01 ^a^	9.29 ± 0.00 ^a^
Chlorogenic acid	29.78 ± 0.01 ^ab^	31.22 ± 0.00 ^a^	28.14 ± 0.01 ^b^	25.33 ± 0.00 ^c^
Ferulic acid	102.59 ± 0.05 ^a^	103.25 ± 0.01 ^a^	107.46 ± 0.02 ^a^	94.13 ± 0.03 ^b^
Synaptic acid	2.56 ± 0.01 ^a^	1.99 ± 0.00 ^ab^	0.95 ± 0.00 ^b^	n.d.

Flavonoids and phenolic acids expressed in μg 100 g^−1^ dry weight (DW). n.d.: Not detected. Results are expressed as mean ± standard deviation. Lowercase superscript letters (a–c) in the same line indicate significant differences among treatments according to Tukey’s test (*p* ≤ 0.05).

**Table 6:** Effect of HIUS and pasteurization on the counts of aerobic mesophilic bacteria, molds and yeasts, and total and thermotolerant coliforms in bright red and yellow passion fruit (*Passiflora edulis* Sims.) pulp.

Parameters	Treatments			
	Control	US-60	US-20	PS-63
**Bright red passion fruit**				
Aerobic mesophilic bacteria (log CFU/mL)	0.00 ± 0.00 ^a^	0.00 ± 0.00 ^a^	0.00 ± 0.00 ^a^	0.00 ± 0.00 ^a^
Molds and yeasts (log CFU/mL)	1.83 ± 0.49 ^a^	1.35 ± 0.49 ^c^	1.69 ± 0.12 ^b^	1.39 ± 0.12 ^c^
Total coliforms (MPN/mL)	<0.3 *	<0.3 *	<0.3 *	<0.3 *
Thermotolerant coliforms (MPN/mL)	<0.3 *	<0.3 *	<0.3 *	<0.3 *
**Yellow passion fruit**				
Aerobic mesophilic bacteria (log CFU/mL)	1.50 ± 0.28 ^a^	2.24 ± 0.14 ^a^	1.72 ± 0.60 ^a^	1.60 ± 0.43 ^a^
Molds and yeasts (log CFU/mL)	2.29 ± 0.20 ^a^	0.00 ± 0.00 ^c^	0.00 ± 0.00 ^c^	1.24 ± 0.34 ^b^
Total coliforms (MPN/mL)	0.4	<0.3 *	<0.3 *	<0.3 *
Thermotolerant coliforms (MPN/mL)	0.4	<0.3 *	<0.3 *	<0.3 *

Results are expressed as mean ± standard deviation. Superscript lowercase letters (a–c) in the same line indicate significant differences among treatments according to Tukey’s test (*p* ≤ 0.05). * Estimated data.

## Data Availability

The original contributions presented in the study are included in the article; further inquiries can be directed to the corresponding author.
